# The Potential of SARMs and Antimyostatin Agents in Addressing Lean Body Mass Loss From GLP‐1 Agonists: A Literature Review

**DOI:** 10.1111/1753-0407.70119

**Published:** 2025-07-31

**Authors:** Jimmy Wen, Ubaid Ansari, Mouhamad Shehabat, Zaid Ansari, Burhaan Syed, Adam Razick, Daniel Razick, Muzammil Akhtar, Eldo Frezza

**Affiliations:** ^1^ California Northstate University College of Medicine Elk Grove California USA; ^2^ University of California, Los Angeles Los Angeles California USA

**Keywords:** antimyostatin, GLP‐1 agonists, lean body mass, literature review, SARMs

## Abstract

Glucagon‐like peptide‐1 receptor agonists (GLP‐1 RAs) have demonstrated substantial weight loss effects among patients with diabetes and obesity. However, given the rapid weight loss induced, there is concern about the total change in body composition, including lean body mass (LBM). Current literature on these effects contains significant heterogeneity, with some studies reporting a loss of 40%–60% of LBM and others reporting 15% or less. To combat this, selective androgen receptor modulators (SARMs) have become a popular candidate. Given their androgen receptor selectivity, SARMs have notable anabolic properties and proposed improved safety profiles over traditional anabolic compounds. Several of these agents, such as enobosarm, have been investigated in clinical trials involving older patient populations or patients with cachexia or sarcopenia secondary to chronic diseases. Furthermore, other agents to maintain or enhance LBM, such as antimyostatin agents, are also under investigation. Exploring this potential synergy could lead to better weight loss and body composition management in patients using GLP‐1 RAs for diabetes or weight loss therapy. This review aims to evaluate the potential benefits and uses of SARMs in ameliorating the body composition changes induced by GLP‐1 RAs. Other investigational agents to retain or increase muscle mass and the future possibilities of these drugs will be discussed.

## Graphical abstract

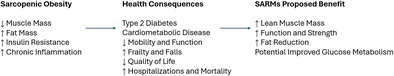

**Summary**

- The combination therapy of SARMs and GLP‐1 RAs can potentially augment fat loss while maintaining or increasing LBM and BMD.
- Other drugs aimed to preserve or enhance LBM and BMD, such as antimyostatin and monoclonals, are also being investigated for cachexia and sarcopenic conditions.
- However, careful analysis of the safety profile of adjuvant therapy must be taken into account, as the drawbacks of additional medication regimens must not outweigh the benefits.

## 1 Introduction

Glucagon‐like peptide‐1 receptor agonists (GLP‐1 RAs) are commonly used for Type 2 diabetes mellitus (T2DM) and obesity. These agents provide glucose‐dependent insulin secretion, feelings of satiety, and reduced proinflammatory cytokines. There is growing evidence of other indications such as cardiovascular risk mitigation, chronic kidney disease management, and conditions that benefit from weight loss such as non‐alcoholic fatty liver disease and obstructive sleep apnea (OSA) [1].

Despite the substantial weight loss induced by GLP‐1 RAs and dual GLP‐1/glucose‐dependent insulinotropic polypeptide (GIP) agonists, there is concern about the total change in body composition, including the effects on lean body mass (LBM). Current reports in clinical trials demonstrate significant heterogeneity in the reported impact on LBM, with some studies reporting a loss of 40%–60%, and others reporting approximately 15% or less [2]. However, the baseline patient demographics across these studies were not standardized for age, different GLP‐1 RAs, dosage, and comorbidities.

It is not clear what the long‐term effects of rapid weight loss induced by GLP‐1 RAs on LBM and muscle function are. Patients who are older and with increased disease severity may be more greatly impacted, given the expected decrease in muscle quality and function commensurate with their comorbidities. Thus, given the potential unfavorable body composition changes induced by GLP‐1 RAs, pharmacological therapies to maintain or increase LBM during GLP‐1 RA administration are currently under development. Selective androgen receptor modulators (SARMs) are anabolic pharmacological agents that were developed to combat skeletal muscle wasting for patients with sarcopenia and cachexia secondary to chronic diseases [3]. SARMs provide similar LBM results as testosterone with a proposed improved safety profile given its purposed androgen receptor selectivity [4]. Despite several clinical trials being conducted with various SARMs, none are currently approved by the Food and Drug Administration (FDA). This review aims to evaluate the potential benefits and uses of SARMs in ameliorating the body composition changes induced by GLP‐1 RAs. Other investigational agents to retain or increase muscle mass and the future possibilities of these drugs will also be discussed.

## 2 Androgen Receptors and GLP‐1 Receptors

Androgen receptors (ARs) are nuclear receptors activated by binding to androgens such as testosterone and dihydrotestosterone (DHT). Upon activation, ARs translocate to the nucleus, where they modulate the expression of genes such as insulin‐like growth factor 1 receptor (IGF‐1R) and cyclin‐dependent kinase 2 (CDK‐2) involved in muscle growth, differentiation, and maintenance [5]. This pathway is critical in maintaining muscle mass and strength, especially in response to growth stimuli such as exercise or injury.

GLP‐1 receptors are G‐protein‐coupled receptors predominantly located in the *α* cells in the pancreas and neurons in the caudal brainstem and hypothalamus [6]. They play a critical role in glucose metabolism by promoting insulin secretion in response to food intake and inhibiting glucagon release [6]. However, a notable side effect of GLP‐1 RAs is muscle loss, possibly due to their influence on body composition by promoting fat loss and potentially affecting protein metabolism [7]. The exact mechanisms by which GLP‐1 agonists contribute to muscle wasting are not fully understood. Growing concern remains regarding their impact on muscle mass, particularly in individuals with limited muscle reserves.

The relationship between ARs and GLP‐1 receptors is not direct, but there may be overlapping metabolic pathways. Both receptors influence body composition—ARs by promoting muscle hypertrophy and GLP‐1 receptors by modulating energy homeostasis and body weight. One study that analyzed male mice showed that the absence of ARs in pancreatic *β* cells leads to reduced insulin secretion in response to glucose, causing hyperglycemia [8]. Testosterone works through extranuclear ARs to amplify the insulin‐stimulating effects of GLP‐1 by enhancing cAMP production via mitochondrial CO_2_ generation and G alpha subunit (Gαs) recruitment. Additionally, testosterone boosts insulin secretion in human islets through a signaling cascade involving actin remodeling, highlighting both genomic and non‐genomic AR actions in regulating insulin release [8]. Testosterone also plays a role in enhancing insulin sensitivity and promoting glucose‐stimulated insulin secretion. Therefore, lower testosterone levels increase the risk of hyperglycemia and diabetes mellitus Type 2 by reducing insulin secretion and impairing glucose metabolism [9].

### 2.1 GLP‐1 Agonists

**FIGURE 1:**
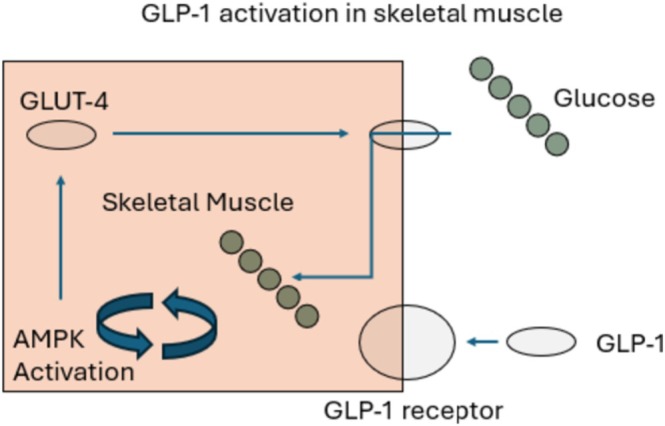
GLP‐1 activation in skeletal muscle.

GLP‐1 RAs are a class of medications that mimic the action of endogenous GLP‐1, a hormone produced in response to food intake. These agents activate the GLP‐1 receptor, leading to increased insulin secretion, decreased glucagon release, delayed gastric emptying, and reduced appetite [10]. The GLP‐1 receptor has a wide range of pharmacological targets in the body, including the brain, pancreas, liver, kidney, skeletal muscle, and adipose tissue [10]. In liver tissue, mouse models have shown that GLP‐1 RAs attenuated the mechanistic target of the rapamycin (mTOR) pathway while enhancing the AMP‐activated protein kinase (AMPK) pathway [6]. This mechanistic activation promotes lipolysis by activating the AMPK pathway and decreases lipogenesis by inhibiting the mTOR pathway. AMPK is also involved in modulating levels of glucose uptake and glycogen synthesis in skeletal muscle [6]. This can be found depicted in Figure 1.

**FIGURE 2:**
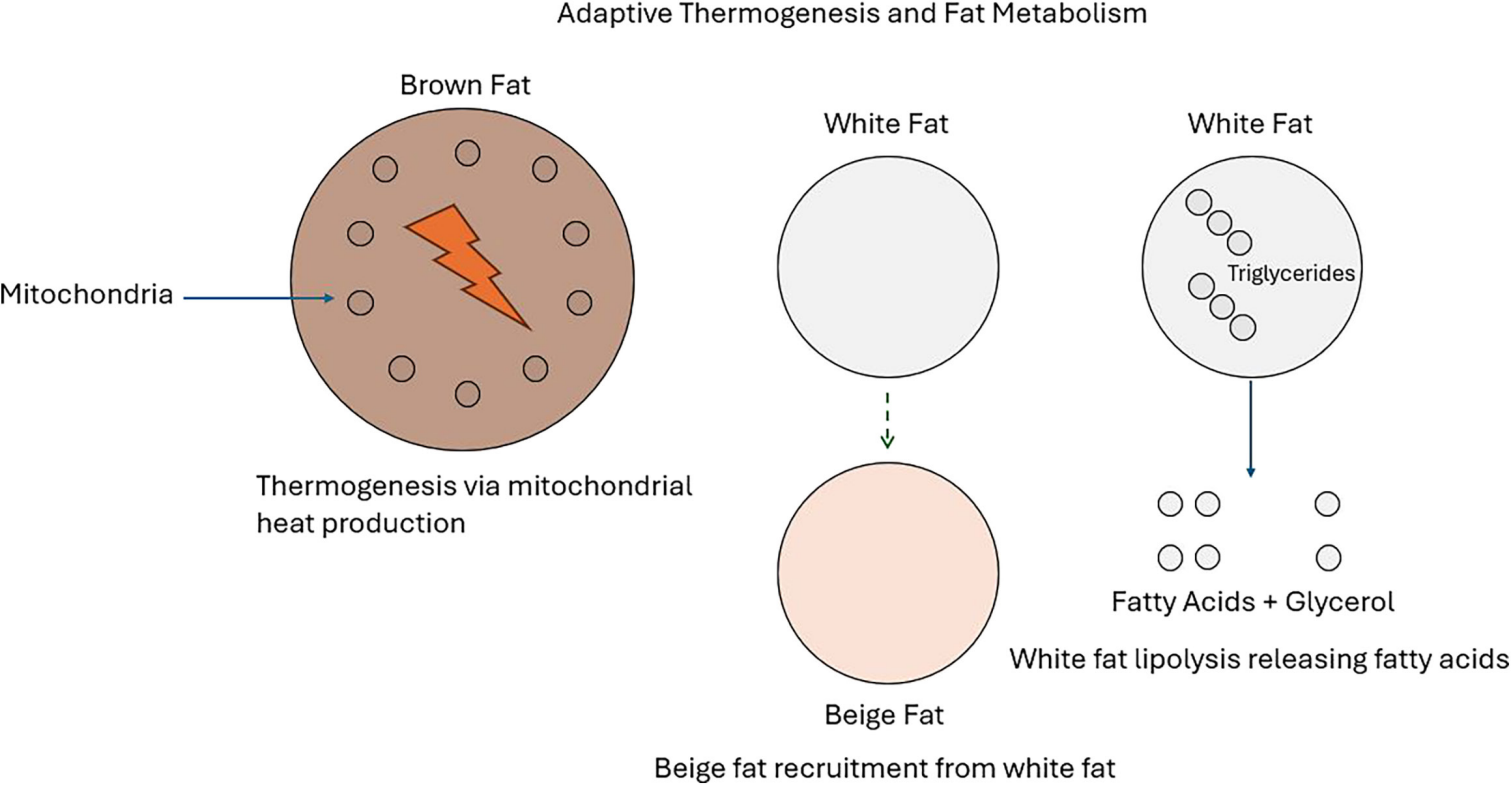
Adaptive thermogenesis and fat metabolism.

Furthermore, mounting evidence shows that GLP‐1, GIP, and glucagon receptors help modulate and promote thermogenesis [10]. Activation of these receptors appears to increase heat production and adaptive thermogenesis via amino acid metabolism and fatty acid oxidation in brown adipose tissue and beige fat, leading to increased energy expenditure and overall weight loss. Thus, GLP‐1, GIP, and glucagon receptor activation may enhance adipose thermogenesis by increasing the activity of existing thermogenic fat or recruiting more thermogenic fats [10]. These effects are illustrated in Figure 2. This multifaceted approach helps improve glycemic control and promotes weight loss, making GLP‐1 RAs a valuable tool in the treatment of T2DM and obesity.

One well‐known GLP‐1 RA is liraglutide (Victoza for T2DM and Saxenda for obesity). Liraglutide has been shown to improve blood sugar control by enhancing insulin release when glucose levels are high, while also reducing postprandial glucagon secretion [11]. It also delays gastric emptying, which contributes to feelings of satiety and aids in weight reduction. Liraglutide has been shown in clinical trials, such as the LEADER trial, to reduce cardiovascular risks in patients with T2DM, highlighting its benefits beyond glucose control [12].

Another prominent GLP‐1 RA is semaglutide (Ozempic for T2DM and Wegovy for obesity), which has gained popularity due to its potent effects on both glycemic control and weight reduction. Semaglutide, administered once weekly, has been shown to significantly reduce HbA1c levels and promote substantial weight loss in individuals with obesity [13]. In the STEP trials, semaglutide demonstrated its efficacy in reducing body weight by up to 15% in some individuals [14]. However, this profound weight loss raises concerns about potential muscle mass loss alongside fat reduction. Although semaglutide's effects are generally seen as positive for metabolic health, preserving muscle mass is critical, especially in populations prone to sarcopenia or muscle wasting [15].

Another example is dulaglutide (Trulicity), which shares similar mechanisms of action and therapeutic benefits with liraglutide and semaglutide. Administered once weekly, dulaglutide has been shown to improve glycemic control and reduce the risk of major cardiovascular events in patients with T2DM [16]. Like other GLP‐1 agonists, dulaglutide's benefits on body composition are primarily focused on reducing fat mass, but concerns remain regarding its long‐term impact on muscle mass in patients undergoing significant weight loss [17]. Dulaglutide consists of a large GLP‐1 molecule conjugated to an Fc fragment of human IgG4, allowing for its long half‐life and once‐weekly dosing schedule [10]. This prolonged half‐life may contribute to greater appetite suppression compared to shorter‐acting others in its class. However, the larger size potentially limits its penetration into tissues that are involved in appetite suppression and energy regulation, thereby decreasing efficacy for weight loss compared to other GLP‐1 RAs [18]. However, further studies are required to substantiate the effects of GLP‐1 RA molecular size on tissue distribution to target areas that modulate its weight loss effects.

Tirzepatide (Mounjaro for T2DM and Zepbound for obesity) is a novel dual agonist that targets both the GLP‐1 and GIP receptors, offering enhanced effects on glucose control and weight loss [19]. In clinical trials such as the SURPASS studies, tirzepatide has been shown to significantly reduce HbA1c and promote greater weight loss compared to GLP‐1 agonists like semaglutide [20]. Patients experienced up to a 20% body weight reduction, making it one of the most effective treatments for T2DM and obesity. However, similar to other GLP‐1 RAs, there are concerns about potential muscle mass loss, especially in patients undergoing substantial weight reduction [21]. This potential downside emphasizes the importance of monitoring and addressing muscle preservation, particularly in vulnerable populations using GLP‐1 RAs for weight management.

Preliminary mice studies with GLP‐1 RAs found that muscle biopsy results demonstrated decreased muscle atrophy and inflammation, improved muscle vasculature, and increased preservation of mitochondria [1]. Human studies have also shown promising results with the maintenance or increase in muscle mass among elderly patients taking GLP‐1 RAs [22, 23]. A 2025 meta‐analysis by Karakasis et al. found that among 22 randomized controlled trials (2258 patients), there were significant reductions in body weight, fat mass, and lean mass [24]. This reduction in lean mass was approximately 25%, but it was determined that the relative lean mass (percentage change from baseline) was unaffected [24]. Among the GLP‐1 RAs included, liraglutide (3.0 mg weekly or 1.8 mg daily)was the only agent that significantly reduced weight without significant reductions in lean mass. However, tirzepatide (15 mg weekly) and semaglutide (2.4 mg weekly) had the greatest reductions in weight and fat mass, but were also associated with the greatest loss of lean mass [24]. There is increasing evidence of utilizing GLP‐1 RAs in the treatment of inflammatory myopathies as they have been shown to reduce atrophic factors, promote myogenic factors, and reduce inflammation and adiposity [1]. Contemporary imaging with magnetic resonance imaging (MRI) has suggested that skeletal muscle changes after GLP‐1 RA usage are adaptive, given the age and disease burden of the patients assessed [1]. Additionally, the findings of improved insulin sensitivity and muscle fat infiltration likewise suggest an improved muscle quality.

### 2.2 SARMs

**FIGURE 3:**
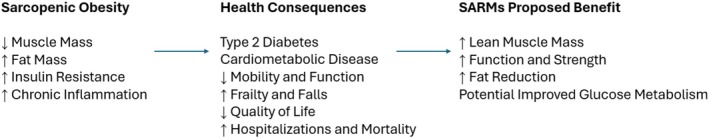
Clinical impact of sarcopenic obesity and therapeutic potential of SARMs.

While GLP‐1 RAs contribute to weight loss, they may inadvertently induce muscle loss in some individuals, creating a potential therapeutic gap that SARMs could fill (Figure 3) [25]. By targeting ARs in muscle tissue, SARMs can potentially offset the muscle loss seen with GLP‐1 RAs without interfering with the positive metabolic effects of GLP‐1 agonists on glucose and fat metabolism.

SARMs are synthetic compounds that resemble androgenic anabolic steroids (AAS)'s anabolic properties but are proposed to bind ARs selectively to decrease the number of adverse effects (AEs) [26]. SARMs are classified as steroidal or nonsteroidal. Nonsteroidal SARMs are not metabolized by 5α‐reductase or aromatase to dihydrotestosterone (DHT) or estrogen, respectively, thus decreasing androgenic activity. Each SARM has its own chemical structure and conformation and can function as a full agonist, partial agonist, or antagonist. However, the exact mechanism is still being debated, with one leading hypothesis being that SARMs induce specific conformational changes in the ligand‐binding domain between the AR and other proteins and transcription factors. These differences in protein–protein interactions and ligand‐specific conformations lead to gene regulation in specific tissues. However, a 2024 systematic review found that at a mean short‐term follow‐up of 80 days, there were 64.7% reported AEs (628/970) [26]. However, there was no significant difference compared to placebo and the majority of patients with these AEs recovered after cessation of the AE. Nevertheless, there are concerning indications for cardiovascular health, with reductions in high‐density lipoprotein (HDL) and increases in hemoglobin and blood pressure [26]. Additionally, there are reports of liver injuries (elevated liver transaminases), rhabdomyolysis, and tendon ruptures [26]. Notably, there was a case reported of a 27‐year‐old male who developed an acute kidney injury and cholestatic liver injury after using multiple SARMs, with kidney biopsy revealing bile casts, consistent with bile cast nephropathy [27]. However, nephrotoxicity reports are currently limited in the literature.

By utilizing the AR pathway, SARMs offer a potential therapeutic approach to counteract muscle loss in various conditions, including those associated with aging, cancer, and possibly medication‐induced muscle wasting [28]. Exploring this potential synergy could lead to better management of muscle loss in patients using GLP‐1 RAs for diabetes or weight loss therapy.

Enobosarm, or ostarine (MK‐2866 or GTx‐024), is a SARM that has recently gained traction for its benefits in increasing muscle mass and combating muscle wasting [29]. Numerous studies have also shown enobosarm to have an increase in general muscle function. In a study done by Dalton et al., in a population of men older than 60 and post‐menopausal women, enobosarm was shown to have a dose‐dependent increase in stair climbing speed, a significant increase in stair climbing power, and a significant decrease in fat mass compared to the placebo [30]. Dobs et al. observed significantly increased total LBM in the enobosarm groups in a population of men older than 45 and postmenopausal women, all of whom have cancer [31].

Given the extensive clinical research performed with enobosarm, the addition of this agent can provide enhanced clinical benefit by mitigating muscle loss with GLP‐1 RAs. It can also augment the fat loss effects of GLP‐1 RAs while preserving or enhancing LBM and bone mineral density (BMD) [27]. For example, in Dalton et al., along with increasing muscle function, it was also found that enobosarm had a significant decrease in blood glucose and insulin resistance, calculated based on the Homeostasis model assessment: insulin resistance, compared to the placebo group. The 3 mg enobosarm group had a reduction of 6.9 mL/dL in blood sugar level and a 2.2 μIU/mL decrease in insulin. Decreases in serum triglycerides, total cholesterol, and high‐density lipoprotein were found as well [30]. With similar functions in GLP‐1 RAs, more studies and trials should be conducted to determine the interaction these drug classes can have. A Phase 2 trial being conducted by Veru is currently evaluating the effect enobosarm has on LBM for patients on semaglutide [32]. Patients will be on semaglutide plus a placebo, 3 or 6 mg enobosarm. The primary endpoint being measured is the percentage change from baseline in total LBM at the end of 4 months. Secondary endpoints will be changes in fat mass, insulin resistance (HOMA‐IR), and body weight at 4 months. Additionally, after the completion of the study, a BMD extension will be performed for an additional 5 months, for a total of 9 months. Safety profiles must also be considered, as GLP‐1 RAs and enobosarm have common AEs in studies, that being nausea, diarrhea, abdominal pain, and constipation [30, 31, 33]. A summary of lean body and fat mass changes in completed and current clinical trials involving enobosarm can be found in Table 1 below.

**TABLE 1:** Current enobosarm clinical trials.

Clinical trial registration number	Phase	Number of patients enrolled	Population	Trial period	Lean body mass changes	Fat mass changes
Protocol G200501	2	120	Males over 60 years of age and postmenopausal women	12 weeks	Placebo: 0.70 kg decrease 0.1 mg: 0.16 kg increase (*p* = 0.474^a^) 0.3 mg: 0.08 kg increase (*p* = 0.651^a^) 1 mg: 0.59 kg (*p* = 0.055^a^) 3 mg group: 1.25 kg increase from baseline (*p* < 0.001^a^)	Placebo: 0.31 kg increase 0.1 mg: 0.22 kg increase (*p* = 0.793^a^) 0.3 mg: 0.07 kg decrease (*p* = 0.242^a^) 1 mg: 0.26 kg decrease (*p* = 0.085^a^) 3 mg: 0.32 kg decrease (*p* = 0.049^a^)
NCT00467844	2b	159	Muscle‐wasting cancer patients	16 weeks	Placebo: 0.1 kg increase (*p* = 0.88^b^) 1 mg Group: 1.5 kg increase (*p* = 0.0012^b^) 3 mg group: 1.30 kg increase (*p* = 0.046^b^)	Placebo: 0.41 kg increase (*p* = 0.272^b^) 0.64 kg decrease (*p* = 0.210^b^) 3 mg: 0.76 kg decrease (*p* = 0.086^b^)
NCT01355484	3	321	Lung cancer muscle wasting receiving cisplatin + taxane chemotherapy	21 weeks	Placebo: 0.92 decrease 3 mg: 0.41 kg increase (*p* < 0.001^a^)	NR
NCT01355497	3	320	Lung cancer muscle wasting receiving cisplati *n* + nontaxane chemotherapy	21 weeks	Placebo: 0.37 kg decrease 3 mg: 0.47 kg increase (*p* < 0.0028^a^)	NR
NCT06282458 (in progress)	2	150 (estimated)	Males over 60 years of age and postmenopausal women, both on semaglutide	16 weeks for lean body and fat mass 28 weeks for fat mass only	NR	NR

^a^ Compared to placebo.

^b^ Compared to baseline.

Several other SARMs are currently undergoing human trials or animal studies, in hopes of evaluating their role as FDA‐approved treatments for cachexia and sarcopenia. The SARMs that have undergone human studies are LGD‐4033, PF‐06260414, GSK2881078, GTx‐024, MK‐0773, and OPK‐88004, and have shown different qualities and effects within the study samples [27]. On the other hand, the SARMs that are still being evaluated via animal studies consist of RAD140 (Testalone), S‐4 (Andarine), S‐23, YK11, SARM 2‐f, and S‐101479. While these drugs may not currently be mainstream treatment modalities, preliminary findings are important to evaluate clinical outcomes, AEs, and differences within this class. The safety profile of SARMs consists of moderate rates of mild and moderate AEs and a low rate of severe AEs even at a short‐term follow‐up [27].

#### 2.2.1 Human Studies

LGD‐4033 was studied in healthy men with a range of dosage from 0.1 to 1 mg across 3 weeks [34]. Its pharmacokinetics consist of a linear pattern and a half‐life of 24–36 h, as well as a dose‐proportional daily administration increase in drug concentration. Findings demonstrated the LBM increased in a dose‐dependent manner, with a significant improvement of 1.21 kg (*p* = 0.047) at the highest dosage (1 mg). Physical strength was also analyzed via 1 repetition maximum (1‐RM) leg press, stair climbing power, and stair climbing speed, and a dose‐related improvement was seen but not significantly different from the placebo trial.

PF‐06260414 was analyzed in healthy adult men with dosages ranging between 1 and 400 mg across 6 weeks [35]. It exhibited a linear pharmacokinetic effect and a half‐life of 7 h, with minimal renal excretion. LBM and other physical parameters were not examined in this study.

Two trials with GSK2881078 were performed, with doses ranging from 0 to 0.75 mg and a follow‐up of 2 and 12.8 weeks with Clark et al. and Mohan et al., respectively [36, 37]. Pharmacokinetic data showed a dose‐proportional increase in exposure and a half‐life greater than 100 h [36]. In terms of LBM, a numerical increase was observed for both males and females [36]. Physical strength was analyzed via 1‐RM leg press, and an increase was observed in men specifically with a mean change from baseline as 11.8 kg (90% CI: −0.5 to 24.0) and the percentage change from baseline between treatment and placebo as 7.0% (90% CI: 0.5–13.6). For women, however, no significant difference was observed, with an adjusted mean change from baseline as 8.0 kg (90% CI: −2.5 to 18.4) and a percentage change from baseline between treatment and placebo as 5.2% (90% CI: −4.7 to 15.0) [37].

MK‐0773 was analyzed with dosages of 50 mg across 26 weeks [38]. A statistically significant increase in LBM was observed in participants compared to placebo, with an increase of 1.26 versus 0.29, respectively (*p* < 0.001). In terms of physical strength, a statistically significant increase was observed in relation to baseline but was not significant compared to placebo. In particular, the bilateral leg press at the 6‐month treatment showed a difference of 5.19 lb (95% CI: −4.04 to 14.43, *p* = 0.269).

OPK‐88004 was analyzed with a duration of 12 weeks with a dosage range from 1 to 15 mg [39]. In terms of LBM, OPK‐88004 exhibited a dose‐dependent significant increase in whole‐body lean mass, appendicular lean mass, and a significant decrease in percentage body fat (*p* < 0.001, for all). Physical performance and other strength assessments showed no significant difference in comparison to the control group.

#### 2.2.2 Animal Studies

RAD140 was tested in an animal study of 23 randomly chosen female mice at a dosage of 5 mg/kg [40]. Muscle strength, adaptability, and overall health effects were measured with the supplementation of RAD140. Results found that RAD140 did not significantly increase muscle strength or muscle wet mass in either basal or stressed states. Muscle adaptability was found to be blunted after eccentric exercises with RAD140's supplementation. Interestingly enough RAD140's supplementation with other SARMS such as LGD‐2226 and GSK2881078, was determined to increase muscle mass as well as strength in both relaxed and stressed conditions [37, 41]. In terms of health and viability, RAD140 supplementation resulted in premature aging in both old and young mice. There also have been several case studies that indicate organ damage with RAD140's supplementation such as liver damage and muscle membrane damage [42, 43]. Further studies are warranted to conclude RAD140s' impact and side effects definitively.

S‐4 was compared to dihydrotestosterone (DHT) in Sprague Dawley rats status post 12 weeks after castration with a dosage of either 3 or 10 mg/kg for 8 weeks [44]. Both S‐4 dosages returned baseline soleus and levator ani muscle mass and strength, which were also seen in the DHT treated rats. However, DHT increased prostate and seminal vesicle size by over two times, while S‐4 stimulated the growth of only 16% and 17%, respectively, compared to the controlled levels. Plasma levels of LH and FSH were decreased in a dose‐dependent manner, as S‐4 was shown to have agonist effects on the pituitary [44]. Thus, S‐4 demonstrated strong anabolic effects in LBM (muscle/bone) and the pituitary without major effects in the prostate and seminal vesicles, suggesting its proposed utility of tissue selectivity.

S‐23 was studied in castrated male rats after 14 days of treatment at doses ranging from 0.01 to 3 mg/day [45]. Like S‐4, S‐23 increased LBM and BMD while reducing fat mass dose‐dependently. However, it was notable that although the size of the prostate decreased, the levator ani muscle increased in size [45].

Morimoto et al. studied 2‐F in rats, and it was found to increase the levator ani muscle by 4.6 and 4.9 fold compared to the control group at 20 and 100 mg/mL doses, respectively [46]. 2‐F also showed enhanced spontaneous locomotive activities in the dark phase in castrated rats but not to the level of non‐castrated rats. 2‐F also had an increase in body weight and food intake in castrated rats, matching those levels in non‐castrated rats. Morimoto et al. studied 2‐F among monkeys and found an increase in body mass from pre‐dosing levels at day 13, with dosages of 1, 3, and 10 mg/kg, by as much as 6.2%, 6%, and 8.7%, respectively. On Day 27, body mass increased by 7.7%, 3.3%, and 11.9%, respectively [47].

## 3 Antimyostatin Agents

Recent developments in the management of muscular atrophy have focused on monoclonal antibodies and agents that target specific pathways involved in attempting to grow and preserve muscle. Among these agents, antimyostatins and other monoclonal therapies have shown promise in preclinical and clinical settings. Myostatin inhibitors have garnered attention as potential therapeutic agents for muscle‐wasting conditions. Myostatin, a member of the transforming growth factor‐beta (TGF‐β) family, negatively regulates muscle growth [48]. Immature myostatin is a 24‐kDa covalent homodimer with a non‐covalent bond formed with its propeptide, remaining as an inactive latent complex. Active myostatin can be released from the latent complex via propeptide cleavage which is found in complexes with inhibitory proteins such as follistatin, follistatin‐like 3, and growth and differentiation factor‐associated serum protein‐1 (GASP‐1) [49]. Its effects are mediated primarily through the activin receptor IIB (ActRIIB) which complexes with activin‐like kinase‐4 (ALK‐4) or ALK‐5. Following this, cytoplasmic receptor‐regulated Smad 2 and 3 are phosphorylated and activated, leading to translocation into the nucleus to induce specific gene changes. Blocking myostatin activity has been proposed to increase muscle mass in various conditions [49, 50]. However, there are still safety concerns related to the long‐term inhibition of myostatin as this pathway plays a complex role in muscle homeostasis. A summary of LBM and fat mass changes in completed clinical trials involving antimyostatin agents can be found in Table 2 below.

**TABLE 2:** Completed clinical trials with antimyostatin agents.

Clinical trial registration number	Phase	Number of patients enrolled	Population	Trial period	Lean body mass changes	Fat mass changes
PF‐06252616 (NCT02841267)	2	19	LGMD2I (limb girdle muscular dystrophy)	32 weeks	NR (records muscle strength)	NR
Bimagrumab (NCT024686740)	2	160	Older adults 50+ with sarcopenia	24 weeks	Placebo (*n* = 5): 32.6 BYM338 700 mg (*n* = 15): 41.4 (8.8 increase) Value = 0.084	NR
Landogrozumab LY2495655 (NCT01369511) (NCT01604408)	2	400	Older patients 50 years + with hip replacement	12 weeks	Placebo (*n* = 74): −0.900 315 mg LY24295655 (*n* = 72): +1.784 (change = 2.684) *p* = 0.007	NR
	2	365	Patients aged 75 years or older who had fallen in the past year	24	Placebo (*n* = 99): −0.123 kg (95% CI: −0.287 to 0.040) LY Group (*n* = 85): 0.303 kg (0.135 to 0.470) +0.43 kg (95% CI: 0.192 to 0.660; *p* < 0·0001)	NR
ACE‐031 (NCT01099761)	2	24	Boys 4+ with duchenne muscular dystrophy	24 weeks	Placebo (*n* = 6): −3.7 ACE‐031 1.0 mg/kg (*n* = 9): −3.3 0.04 change, (*p* = 0.5) insignificant change in muscle score	NR

### 3.1 Bimagrumab

Bimagrumab is a human monoclonal antibody that targets the activin type II receptors (ActRII), which play a central role in regulating skeletal muscle mass [51]. By inhibiting these receptors, bimagrumab blocks the actions of myostatin and other negative muscle growth regulators. Clinical trials have demonstrated its potential to increase LBM and muscle strength, particularly in conditions like sarcopenia and disuse atrophy [52]. In patients with sporadic inclusion body myositis (sIBM), a progressive muscle‐wasting disease, bimagrumab has been shown to improve muscle mass [53]. Eight weeks after dosing, the bimagrumab‐treated patients increased LBM (+5.7% compared with placebo, *p* = 0.014). A recent study explored the therapeutic potential of bimagrumab in diet‐induced obese mice, specifically in combination with the GLP‐1 RA semaglutide. While semaglutide alone resulted in a loss of both fat and LBM, the study found that combination therapy with bimagrumab not only increased LBM (~10%) but also enhanced fat loss compared to semaglutide alone [54] (Table 3).

**TABLE 3:** Ongoing clinical trials with antimyostatin agents.

Clinical trial registration number	Phase	Number of patients enrolled	Population	Trial period	Primary outcomes	Secondary outcomes
Taldefgrobep Alfa (NCT05337553)	3	269	Spinal muscular atrophy	48 weeks	Change in MFM‐32 score	N/A
Bimagrumab (NCT05616013)	2	507	Obese patients	48–64 weeks	Change from baseline in body weight	Change from baseline in (waist circumference, total body fat mass, total body muscle mass)
GYM329 (NCT05548556)	2	48	Facioscapulohumeral muscular dystrophy (FSHD)	52 weeks	Percent change from baseline in contractile muscle volume (CMV)	Change from: baseline in serum concentration of myostatin, fat fraction of muscle
Apitegromab (NCT05156320)	3	188	Spinal muscular atrophy	52 weeks	Change from baseline in (HFMSE) total score	Latent myostatin, (RULM) total score

### 3.2 Landogrozumab (LY2495655)

Landogrozumab inhibits the myostatin protein, which subsequently blocks the myostatin signaling pathway that is a negative regulator of muscle growth and development. A study in older adults 75 years or older with previous falls demonstrated that while landogrozumab increased lean muscle mass at 24 weeks, the least‐squares mean change in aLBM was −0.123 kg (95% CI: −0.287 to 0.040) in the placebo group and 0.303 kg (0.135 to 0.470) in the LY group, a difference of 0.43 kg (95% CI: 0.192 to 0.660; *p* < 0·0001) [55]. Another clinical trial found that LY2495655 with patients 50 years or older with recent hip replacements demonstrated significant increases in LBM (Placebo [*n* = 74]: −0.900, 315 mg LY2495655 [*n* = 72]: 0.585, *p*‐value = 0.031) [56]. Preliminary findings for landogrozumab show the potential to preserve muscle mass in patients with muscle‐wasting diseases, major procedures, and fall injuries. However, further research is needed to validate these findings in larger, longer‐term trials.

### 3.3 ACE‐031

ACE‐031 is a fusion protein and soluble form of ACTRII type B that inhibits myostatin by blocking the activin receptor that binds myostatin. In healthy postmenopausal women, ACE‐031 showed a statistically significant increase in lean muscle mass (3.3%; *p* = 0.03, by DXA) [57]. More early‐phase clinical trials, including those with Duchenne muscular dystrophy (DMD), indicated improvements in muscle mass; however, the clinical development of ACE‐031 in this case was halted due to concerns over side effects, including epistaxis and telangiectasias [58]. Despite these setbacks, the potential therapeutic utility of ACE‐031 and similar agents continues to attract research interest.

### 3.4 PF‐06252616

PF‐06252616 (Domagrozumab) is a humanized recombinant myostatin‐targeting monoclonal antibody. PF‐06252616 was able to induce body weight, muscle weight, and LBM in normal and mdx (mouse model of DMD) mice, and non‐human primates [59]. Two clinical trials were subsequently conducted for its potential to treat muscle‐wasting disorders [60]. In these studies, PF‐06252616 was expected to increase muscle mass; however, they failed to meet significant endpoints related to muscle function improvements, leading to the discontinuation of the development of this drug [61].

### 3.5 Apitegromab

Apitegromab is another promising myostatin inhibitor that specifically targets the precursor/inactive form of myostatin. It gained particular attention for its application in patients with spinal muscular atrophy (SMA). Crawford et al. conducted a study with three cohorts (ambulatory patients aged 5–21 with apitegromab 20 mg/kg alone or in combination with nusinersen, nonambulatory patients aged 5–21 years with apitegromab 20 mg/kg combined with nusinersen, and apitegromab, 2 or 20 mg/kg, combined with nusinersen in patients ≥ 2 years). Utilizing the Hammersmith Functional Motor Scale, at month 12 the mean change from baseline for cohorts 1–3 were −0.3 points (95% CI: −2.1 to 1.4), 0.6 points (95% CI: −1.4 to 2.7), 5.3 (−1.5 to 12.2) and 7.1 (95% CI: 1.8 to 12.5), respectively [62]. Apitegromab has demonstrated the ability to enhance motor function in patients receiving SMN‐enhancing therapies, providing a synergistic effect on muscle preservation [62].

### 3.6 Taldefgrobep

Taldefgrobep is a novel myostatin and activin A inhibitor that has shown promise in clinical trials for muscle‐wasting conditions. Similar to other antimyostatins, taldefgrobep works by inhibiting the negative regulatory effects of myostatin on muscle growth. Muntoni et al. reported the cumulative data across three trials of taldefgrobep, including 1 Phase 1 study with adult healthy volunteers and two randomized controlled trials in ambulatory boys with DMD (Phase 1b/2 and Phase 2/3) [63]. In the Phase 1 study, taldefgrobep produced a significant increase in thigh muscle volume and a dose‐dependent suppression of myostatin. In the Phase 1b/2 trial, LBM increased dose‐dependently, but the effects on muscle were not as substantial. Finally, the Phase 2/3 trial did not achieve the primary endpoint of functional change for boys with DMD using the North Star Ambulatory Assessment total score. However, this agent was well tolerated, and no patients withdrew as a result of treatment [63].

### 3.7 GYM329

GYM329 is an investigational antimyostatin agent, similar to apitegromab by binding the latent version of myostatin. Preliminary data suggests that GYM329 has a strong potential for increasing lean muscle mass and improving muscle function in patients with various muscular atrophies. Additionally, GYM329 uses “sweeping antibody technology” to reduce myostatin levels in the muscle and plasma [64]. The same study compared GYM329 with conventional antimyostatin agents in three different mouse disease models and found muscle strength‐improvement effects in all of them. Finally, it was shown that the GYM329 surrogate increased muscle mass in normal cynomolgus monkeys with no obvious toxicity [64]. This has spurred the movement into ongoing Phase 2 trials listed below. As GYM329 is still in early‐phase trials, further studies are necessary to evaluate its long‐term impact on muscle strength, functional outcomes, and overall safety.

## 4 Conclusion

SARMs have been investigated among both older populations and in patients with cachexia and sarcopenia secondary to chronic diseases. However, the long‐term safety profile of SARMs has not been elucidated. Even at a short follow‐up, SARMs exhibit a notable amount of AEs. Nonetheless, the combination therapy of SARMs and GLP‐1 RAs can potentially augment fat loss while maintaining or increasing LBM and BMD. Other drugs aimed at preserving or enhancing LBM and BMD, such as antimyostatin and monoclonals, are also being investigated for cachexia and sarcopenic conditions. However, careful analysis of the safety profile of adjuvant therapy must be taken into account as the drawbacks of additional medication regimens must not outweigh the benefits. Future research with GLP‐1 RAs should focus on developing more accurate measurements of muscle strength, function, and overall body composition to better determine the impact of these agents on LBM. Additionally, combination therapies with SARMs or other muscle‐preserving agents should be evaluated with GLP‐1 RAs to examine the possible synergistic effects.

## Author Contributions

J.W., A.R., D.R., M.A., and E.F. contributed to conceptualization and design, and supervision. J.W., U.A., M.S., Z.A., B.S., A.R., D.R., and M.A. contributed to data acquisition and data analysis.

## Conflicts of Interest

The authors declare no conflicts of interest.

## Data Availability

No new datasets were generated for this study.
